# 骨髓增生异常肿瘤诊断与治疗中国指南（2026年版）

**DOI:** 10.3760/cma.j.cn121090-20251205-00574

**Published:** 2026-02

**Authors:** 中华医学会血液学分会

## Abstract

骨髓增生异常肿瘤（MDS）是一组起源于造血干细胞的高度异质性髓系肿瘤，临床表现为一系或多系血细胞减少，有向急性髓系白血病转化的风险。近年来，MDS在基础研究和新型靶向药物治疗方面取得了较大进展，第五版WHO分类对MDS的分型进行了更新。因此，中华医学会血液学分会对《骨髓增生异常综合征中国诊断与治疗指南（2019年版）》进行了修订，以规范MDS的诊断、鉴别诊断、预后评估及治疗选择，从而更好地指导临床实践。

## Abstract (en)

Myelodysplastic neoplasms（MDS）are a group of highly heterogeneous myeloid tumors originating from hematopoietic stem cells, clinically characterized by single or multiple lineage cytopenia with a risk of acute myeloid leukemia transformation. In recent years, significant progress has been made in basic research and the development of novel targeted therapy for MDS, and the 5th edition of the WHO classification has updated the subtypes of MDS. Therefore, the Chinese Society of Hematology, Chinese Medical Association has revised the “Chinese Guidelines for Diagnosis and Treatment of Myelodysplastic Syndromes（2019）” to standardize the diagnosis, differential diagnosis, prognostic assessment, and treatment selection for MDS, thereby better guiding clinical practice.

骨髓增生异常肿瘤（myelodysplastic neoplasms，MDS），此前称骨髓增生异常综合征（myelodysplastic syndromes，MDS），是一组起源于造血干细胞的恶性克隆性疾病，其特点是一系或多系血细胞减少、髓系各阶段细胞发育异常和高风险向急性髓系白血病（acute myeloid leukemia，AML）转化。2014–2018年间，经年龄调整的MDS每年的发病率为4.2/10万。MDS好发于老年人群，发病率随着年龄的增长显著升高，诊断时的中位年龄为77岁，小于50岁患者的比例不足10％[1]。近年来，MDS的临床研究取得了较大进展，为进一步提高我国医务人员对MDS的认识和诊治水平，中华医学会血液学分会在《骨髓增生异常综合征中国诊断与治疗指南（2019年版）》[2]的基础上制订了本版指南。

一、诊断标准与鉴别诊断

MDS的最低诊断标准见表1。按照世界卫生组织（World Health Organization，WHO）的标准，血细胞减少的标准为：中性粒细胞绝对计数（absolute neutrophil count，ANC）<1.8×10^9^/L，血红蛋白（HGB）<130 g/L（男）或<120 g/L（女），血小板计数（PLT）<150×10^9^/L。

**表1:** 2016年骨髓增生异常肿瘤（MDS）国际工作组建议的MDS最低诊断标准[3]

MDS诊断需满足2个必要条件和1个主要标准
（1）必要条件（2条均须满足）
①持续≥4个月一系或多系血细胞减少（如检出原始细胞增多或MDS相关细胞遗传学异常，无需等待可诊断MDS）
②排除其他可导致血细胞减少和发育异常的造血及非造血系统疾病
（2）MDS相关（主要）标准（至少满足1条）
①发育异常：骨髓涂片中红细胞系、粒细胞系、巨核细胞系发育异常细胞的比例≥10％
②环状铁粒幼细胞占有核红细胞比例≥15％，或≥5％且同时伴有SF3B1突变
③原始细胞：骨髓涂片5％～19％或外周血涂片2％～19％
④常规核型分析或FISH检出有MDS诊断意义的染色体异常
（3）辅助标准（对于符合必要条件、未达主要标准、存在输血依赖的大细胞性贫血等常见MDS临床表现的患者，如符合≥2条辅助标准，诊断为疑似MDS）
①骨髓活检切片的形态学或免疫组化结果支持MDS诊断
②骨髓细胞的流式细胞术检测发现多个MDS相关的表型异常，并提示红系和（或）髓系存在单克隆细胞群
③基因测序检出MDS相关基因突变，提示存在髓系细胞的克隆群体

MDS的诊断属于排除性诊断，应首先排除可能发展为MDS的前驱疾病，包括克隆性造血（clonal hematopoiesis，CH）、潜能未定克隆性造血（clonal hematopoiesis of indeterminate potential，CHIP）、意义未明特发性血细胞减少（idiopathic cytopenia of undetermined significance，ICUS）以及意义未明克隆性血细胞减少（clonal cytopenia of undetermined significance，CCUS）等[1],[4]。

CH指携带获得性突变的造血干细胞克隆性扩增，其发生率随年龄增长而增加，与全因死亡率、心血管疾病及髓系肿瘤风险相关，如VEXAS（空泡、E1酶、X连锁、自身炎症性、体细胞突变）综合征体现了炎症和CH或髓系肿瘤之间的相互作用[5]。第五版WHO分型将CH视为髓系前体细胞损伤。CHIP是CH的主要亚型，特指检测到变异等位基因频率（variant allele frequency，VAF）≥2％（男性X连锁基因≥4％）的髓系肿瘤相关基因突变，但无血细胞减少或可诊断的血液疾病。ICUS指患者在缺乏克隆性证据的情况下存在持续不明原因的血细胞减少，检测不到血液肿瘤相关驱动基因。若CHIP患者出现持续性不明原因血细胞减少，且不符合髓系肿瘤诊断标准，则称为CCUS。

其他常见需要与MDS鉴别的因素或疾病包括：

1. 先天性或遗传性血液病：如先天性红细胞生成异常性贫血、遗传性铁粒幼细胞性贫血、先天性角化不良、范可尼贫血、先天性中性粒细胞减少症和先天性纯红细胞再生障碍等。

2. 其他累及造血干细胞的疾病：如再生障碍性贫血、阵发性睡眠性血红蛋白尿症、原发性骨髓纤维化、大颗粒淋巴细胞白血病、急性白血病等。

3. 巨幼细胞贫血：MDS患者细胞形态发育异常可见巨幼变，易与巨幼细胞贫血混淆，但后者是由叶酸、维生素B_12_缺乏所致。

4. 接受细胞毒性药物、细胞因子治疗或接触有血液毒性的化学制品或生物制剂等。

5. 慢性病性贫血（感染、非感染性疾病或肿瘤）、慢性肝病、慢性肾功能不全、病毒感染（如HIV、巨细胞病毒、EB病毒等）。

6. 自身免疫性血细胞减少、甲状腺功能减退或其他甲状腺疾病。

7. 重金属（如砷剂等）中毒、过度饮酒、铜缺乏。

二、分型

第五版WHO分型标准保留既往形态学病态造血标准，但进一步强调遗传学在MDS发病、诊断和预后中的重要作用，新提出以遗传学和形态学来分型，明确遗传学改变分型——MDS-5q、MDS-SF3B1和MDS-biTP53。新版分型去掉了基于病态造血系列的分型，统一归为MDS伴低原始细胞（MDS with low blasts，MDS-LB）；在MDS-LB的基础上新增了低增生性MDS（MDS hypoplastic，MDS-h）；以MDS伴原始细胞增多（MDS with increased blasts，MDS-IB）代替了前一版的MDS-EB；关注骨髓纤维化的重要性，在MDS-IB中新增了MDS-f。第五版WHO关于MDS的分型及定义见表2。

**表2:** 世界卫生组织（WHO）第五版骨髓增生异常肿瘤（MDS）分型

分型	原始细胞	细胞遗传学	基因突变
MDS伴特定遗传学异常			
MDS伴低原始细胞和孤立5q缺失（MDS-5q）	骨髓<5％且外周血<2％	孤立5q−，或伴除−7和7q−以外的1个核型异常	–
MDS伴低原始细胞和SF3B1突变（MDS-SF3B1）^a^	骨髓<5％且外周血<2％	不伴5q−、−7或复杂核型	SF3B1
MDS伴TP53双等位基因改变（MDS-biTP53）	骨髓和外周血<20％	通常为复杂核型	≥2个TP53位点突变，或单一突变伴TP53拷贝丢失、拷贝中性杂合性缺失
形态学定义的MDS			
MDS伴低原始细胞（MDS-LB）	骨髓<5％且外周血<2％	–	–
MDS，骨髓低增生性（MDS-h）^b^	–	–	–
MDS伴原始细胞增多（MDS-IB）			
MDS-IB1	骨髓5％～9％或外周血2％～4％	–	–
MDS-IB2	骨髓10％～19％或外周血5％～19％或出现Auer小体	–	–
MDS伴骨髓纤维化（MDS-f）	骨髓5％～19％或外周血2％～19％	–	–

**注** SF3B1：剪接因子3b亚基1；^a^环状铁粒幼细胞≥15％可替代SF3B1突变，命名为“MDS伴低原始细胞和环状铁粒幼细胞”；^b^定义为骨髓增生≤25％（根据年龄调整）；–：无内容

三、诊断方法

MDS的诊断基于外周血血细胞计数、骨髓和外周血涂片细胞形态学、免疫分型、细胞遗传学及分子生物学检测等，诊断时建议进行骨髓活检。

1. 血象和骨髓象：约半数以上MDS患者表现为全血细胞减少。一系减少少见，多为红细胞减少。骨髓多增生活跃或明显活跃，少数病例可增生减低。病态造血的特征与MDS的生物学特性及典型遗传学改变有关，病态造血的类型归纳于表3。

**表3:** 骨髓增生异常肿瘤的形态学异常

红系	粒系	巨核系
细胞核 核出芽 核间桥 核碎裂 多核 巨幼样变	细胞核 核分叶减少（假性佩-许畸形） 不规则核分叶增多	细胞核 小巨核细胞 核少分叶 多核（正常巨核细胞为单核分叶）
细胞质 环状铁粒幼细胞 空泡 PAS染色阳性	细胞质 细胞体积过小 颗粒减少或无颗粒 假Chediak-Higashi颗粒 Auer小体	

2. 骨髓病理检测：所有怀疑为MDS的患者均应行骨髓活检，骨髓活检细胞学分析有助于排除其他可能导致血细胞减少的因素或疾病，并提供骨髓细胞增生程度、巨核细胞数量、原始细胞比例、骨髓纤维化程度及肿瘤骨髓转移等重要信息。怀疑为MDS的患者应行Gomori银染色和原位免疫组化（immunohistochemistry，IHC），常用的检测标志包括CD34、MPO、GPA、CD61、CD42、CD68、CD20和CD3。常见病理学异常包括：红系形态及定位异常，巨核细胞胞体大小不等，核叶多变，可为单叶、双叶或多叶，可见单圆核巨核细胞及小巨核细胞；骨髓网硬蛋白纤维增生。

3. 细胞遗传学检测：40％～60％的MDS患者可检出非随机的染色体异常，以−5/5q−、−7/7q−、+8、20q−和−Y最为多见。针对MDS常见异常的组套探针进行荧光原位杂交（fluorescence in situ hybridization，FISH）检测，有助于提高部分MDS患者细胞遗传学异常检出率，通常探针包括：5q31、CEP7、7q31、CEP8、20q、CEPY和TP53。MDS患者常见细胞遗传学异常见表4。

**表4:** 初诊骨髓增生异常肿瘤（MDS）患者常见重现性遗传学异常

不平衡^a^	平衡^b^
−7/del（7q）^c^	t（11;16）（q23.3;p13.3）
−5/del（5q）^c^	t（3;21）（q26.2;q22.1）
i（17q）/t（17p）^c^	t（1;3）（p36.3;q21.1）
−13/del（13q）	t（2;11）（p21;q23）
del（11q）	inv（3）（q21.3;q26.2）/t（3;3）（q21.3;q26.2）
del（12p）/t（12p）	t（6;9）（p23;q34.1）
del（9q）	
idic（X）（q13）	
del（20q）	
+8^c^	
−Y	

**注** ^a^如果无法进行染色体核型分析，FISH的最低推荐组合包括^c^标记的异常及针对TP53的FISH。如没有病态造血，−13或del（13q）、del（9q）等细胞遗传学异常不能用于诊断MDS，而应采用与潜能未定克隆性造血突变相同的方法处理。^b^根据第五版WHO对急性髓系白血病的诊断标准，伴有此类细胞遗传学异常，原始细胞≤20％诊断为急性髓系白血病

4. 免疫表型：流式细胞术可用于量化CD34^+^和CD117^+^髓系祖细胞的比例，还能可靠地识别原始细胞和非原始细胞的异常特征，有助于支持MDS的诊断。欧洲白血病网/国际MDS流式细胞术工作组提出了应用流式细胞术诊断MDS的建议[6]，可以根据评分不同程度地支持MDS的诊断。

5. 分子遗传学检测：80％～90％的MDS患者存在基因突变。早在2017年，国内学者第一次利用二代测序揭示了我国MDS患者的基因突变谱[7]，另有国内学者发现携带der（1;7）（q10;p10）的MDS患者中，RUNX1基因突变的发生频率非常高[8]。我国学者还发现，伴DDX41突变髓系肿瘤患者去甲基化治疗的缓解率达69％，两年总生存（OS）率达85％[9]。MDS国际工作组建议，基因突变监测应至少包括16个预后基因形成的17种突变类型（ASXL1、CBL、DNMT3A、ETV6、EZH2、FLT3、IDH2、KRAS、MLLPTD、NPM1、NRAS、RUNX1、SF3B15q、SF3B1α、SRSF2、TP53多重打击和U2AF1）。50岁以下患者建议加做胚系突变检测。建议MDS患者检测的体细胞突变和易感的胚系突变基因见表5。

**表5:** 骨髓增生异常肿瘤（MDS）相关突变基因列表

基因突变类型	MDS相关突变基因
常见基因突变	DNMT3A、TET2、ASXL1、JAK2、TP53、SF3B1、PPM1D、SRSF2、IDH1、IDH2、U2AF1、KRAS、NRAS、CTCF、CBL、GNB1、BRCC3、PTPN11、GNAS、BCOR、BCORL1
其他基因突变	BRAF、CALR、CEBPA、CREBBP、CSF1R、CSF3R、CUX1、ETV6、EZH2、GATA2、JAK3、KDM6A、KIT、KMT2A、MPL、MYD88、NOTCH1、PHF6、PIGA、PRPF40B、PTEN、RAD21、RUNX1、SETBP1、SF1、SF3A1、SMC1A、SMC3、STAG2、STAT3、U2AF2、WT1、ZRSR2

四、预后分层

目前国内外常用的MDS疾病危险度分层的预后评分系统包括国际预后积分系统（International prognostic scoring system，IPSS）（表6）、WHO预后积分系统（WHO-based prognostic scoring system，WPSS）及修订国际预后积分系统（Revised international prognostic scoring system，IPSS-R）（表7）。国际MDS预后工作组（International Working Group for Prognosis in MDS，IWG-PM）建立了基于基因突变的预后风险模型[10]，即MDS分子国际预后评分系统（Molecular International Prognosis Scoring System，IPSS-M）。该系统沿用了IPSS-R系统中的部分临床参数（包括骨髓的原始细胞比例、PLT及HGB）和细胞遗传学的危险度分组，同时筛选出31个基因（其中16个为预后相关基因形成的17种突变类型，另外15个为其他基因组）（表8）。每种突变类型被赋予不同的数据模型权重进行评分。为了简化IPSS-M的临床使用，研究开发了一个开放访问的网络版评分系统（https://mds-risk-model.com）。而国内MDS患者与国外IWG-PM队列存在以下差异：中位发病年龄更低、血细胞减少程度更严重、骨髓原始细胞增多及不良染色体核型比例增高。国内研究队列表明，IPSS-M在年龄>60岁的MDS患者中预测效力更强，提示IPSS-M的风险评估需整合年龄等相关因素以优化预测效能[11]。

**表6:** 骨髓增生异常肿瘤的国际预后积分系统（IPSS）

预后变量	积分				
	0	0.5	1	1.5	2
骨髓原始细胞（％）	≤5	5～10	–	11～20	21～30
染色体核型^a^	好	中等	差	–	–
血细胞减少系列^b^	0～1	2～3	–	–	–

**注** ^a^预后好核型：正常，−Y，del（5q），del（20q）；预后中等核型：其余异常；预后差核型：复杂（≥3个异常）或7号染色体异常。^b^中性粒细胞绝对计数<1.8×10^9^/L，HGB<100 g/L，PLT<150×10^9^/L。IPSS危险度分类：低危：0分；中危-1：0.5～1分；中危-2：1.5～2分；高危：≥2.5分；–：不适用

**表7:** 骨髓增生异常肿瘤修订国际预后积分系统（IPSS-R）

预后变量	积分						
	0	0.5	1	1.5	2	3	4
细胞遗传学^a^	极好	–	好	–	中等	差	极差
骨髓原始细胞（％）	≤2	–	>2～<5	–	5～10	>10	–
血红蛋白（g/L）	≥100	–	80～<100	<80	–	–	–
血小板计数（×10^9^/L）	≥100	50～<100	<50	–	–	–	–
中性粒细胞绝对计数（×10^9^/L）	≥0.8	<0.8	–	–	–	–	–

**注** ^a^极好：−Y，del（11q）；好：正常核型，del（5q），del（12p），del（20q），del（5q）附加另一种异常；中等：del（7q），+8，+19，i（17q），其他1个或2个独立克隆的染色体异常；差：−7，inv（3）/t（3q）/del（3q），−7/del（7q）附加另一种异常，复杂异常（3个）；极差：复杂异常（>3个）。IPSS-R危险度分类：极低危：≤1.5分；低危：>1.5分且≤3分；中危：>3分且≤4.5分；高危：>4.5分且≤6分；极高危：>6分。–：不适用

**表8:** 骨髓增生异常肿瘤分子国际预后评分系统（IPSS-M）中的基因突变

基因类型	突变类型
主要效应基因（16个）	TP53^multi^、MLL^PTD^、FLT3、SF3B1^5q^、NPM1、RUNX1、NRAS、ETV6、IDH2、CBL、EZH2、U2AF1、SRSF2、DNMT3A、ASXL1、KRAS、SF3B1^α^
其他基因（15个）	BCOR、BCORL1、CEBPA、ETNK1、GATA2、GNB1、IDH1、NF1、PHF6、PPM1D、PRPF8、PTPN11、SETBP1、STAG2、WT1

五、治疗

（一）最佳支持治疗

无论患者的诊断分型、风险评分如何，对症治疗应贯穿始终。对症支持治疗的主要目标为提升患者生活质量，包括成分输血、预防及抗感染、去铁治疗。

1. 成分输血：症状性贫血患者可以输注红细胞改善贫血症状。伴有出血症状的血小板减少患者输注血小板。无活动性出血的患者，推荐PLT<10×10^9^/L时予输注血小板。

2. 预防及抗感染：不建议常规预防感染，但患者开始接受高强度治疗时可以预防性应用。G-CSF/GM-CSF推荐用于中性粒细胞缺乏且伴有反复或持续性感染的MDS患者，不建议持续使用。对于合并感染的患者予积极的抗感染治疗。

3. 去铁治疗：慢性铁过载可对肝脏、心脏及内分泌功能尤其是胰腺功能产生不良影响。对于输血依赖的患者应定期监测血清铁蛋白（SF）水平、累计输血量和器官功能（心脏、肝脏、胰腺），评价铁过载程度（有条件的单位可采用MRI评估心脏和肝脏的铁沉积程度）。对于预期寿命≥1年、输注红细胞总量≥20 U、SF≥1 000 ng/ml至少2个月及输血依赖的患者，可实施去铁治疗，将SF降至1 000 ng/ml以下。常用的去铁药物有去铁胺和地拉罗司等。去铁胺常见的不良反应包括恶心，关节痛，肌痛，发热及注射部位疼痛、肿胀、红斑、瘙痒等，部分患者可出现肌酐升高，甚至急性肾功能衰竭。地拉罗司应用于输血依赖的MDS及中低危MDS合并铁过载，其常见的不良反应包括中性粒细胞减少和血小板减少，通常为轻度或中度。部分患者可出现肝肾功能异常，严重者可出现急性肾衰竭或肝衰竭及消化道出血。对于肌酐清除率<40 ml/min的患者，避免应用去铁胺及地拉罗司。

（二）较低危组MDS的治疗

根据现有预后评分系统，较低危组包含IPSS评分≤1分的低危组/中危-1组、IPSS-R评分≤3.5分的极低危组/低危组/中危组及IPSS-M评分≤0分的极低危组/低危组/中低危组。较低危组MDS的治疗目标是改善血象、提升患者生活质量，其治疗路径见图1。

**图1:**
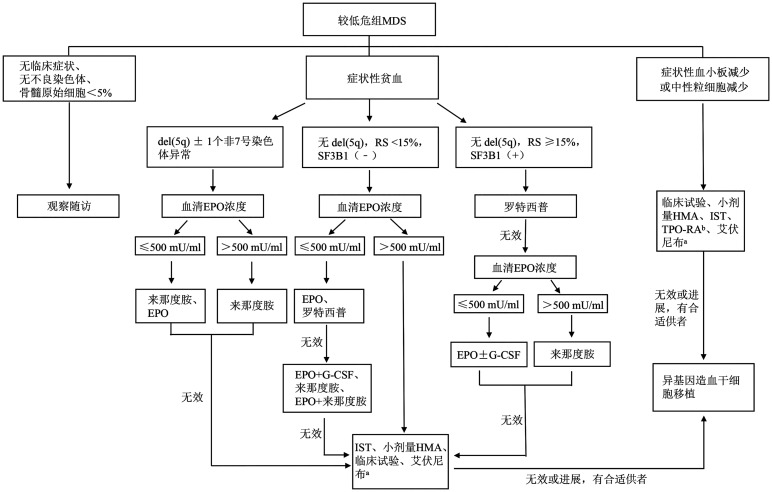
较低危组骨髓增生异常肿瘤（MDS）的治疗路径 **注** ^a^艾伏尼布仅适用于伴IDH1突变患者；^b^TPO-RA用于严重的血小板减少且骨髓原始细胞<5％的较低危MDS，用药过程关注疾病进展风险。RS：环状铁粒幼细胞；EPO：促红细胞生成素；G-CSF：粒细胞集落刺激因子；TPO：血小板生成素；TPO-RA：TPO受体激动剂；IST：免疫抑制治疗；HMA：去甲基化治疗

1. 促造血治疗：

（1）人促红细胞生成素（hEPO）：对于存在症状性贫血或输血依赖的非del（5q）患者，hEPO应尽早使用以获得最大疗效。推荐起始剂量为每周40 000 U，连续治疗8周。若效果不佳，建议第9周起加量至每周60 000 U，继续治疗8周。对于红系应答的患者，hEPO剂量为维持应答所需要的最小剂量。若hEPO每周60 000 U红系应答仍不佳，采用EPO+G-CSF治疗8周评估疗效。hEPO治疗完成24周后，若红系应答不良，判断为hEPO治疗失败。

（2）罗特西普：是一种可溶性融合蛋白，能结合转化生长因子β（TGF-β）配体，减少SMAD2和SMAD3信号传导，从而促进幼红细胞向晚期红细胞的分化成熟。在较低危MDS中可改善红细胞的输血依赖，提升HGB水平[12]。罗特西普的安全性及有效性也在亚洲人群中得到证实[13]。罗特西普为环状铁粒幼细胞（RS）≥15％或RS≥5％伴SF3B1突变的较低危组MDS患者贫血治疗的首要选择。推荐罗特西普起始用量为每次1 mg/kg，每3周为1个疗程，后续根据治疗反应调整剂量。治疗2个疗程后若患者未脱离输血依赖，罗特西普加量至1.33 mg/kg，继续治疗2个疗程。若持续效果不佳，可调整为最大剂量1.75 mg/kg，每3周为1个疗程。罗特西普最常见的不良反应包括疲劳、腹泻、气喘、恶心和头晕，这些不良反应随着时间的推移而减少。3级以上的不良反应有骨痛及高血压，少数患者有发生静脉血栓的风险。目前我国批准罗特西普用于需要定期输注红细胞的极低危、低危和中危成人MDS的贫血治疗。

（3）TPO受体激动剂（TPO-RA）：包括艾曲泊帕、海曲泊帕、阿伐曲泊帕、芦曲泊帕及罗普司亭。TPO-RA可以减少较低危MDS患者的出血风险。对于严重单一血小板减少的患者，若骨髓原始细胞<5％，可考虑单独使用TPO-RA。但需要关注TPO-RA导致的MDS疾病进展风险，需谨慎用药。目前TPO-RA尚未在MDS血小板减少患者中获批适应证。

（4）其他治疗方案：全反式维甲酸（ATRA）联合hEPO和十一酸睾酮治疗低危MDS患者，可改善红系应答及输血依赖[14]。罗特西普单药治疗复发难治低危MDS，可改善红系应答反应[15]。

2. 免疫调节治疗：常用的免疫调节药物为来那度胺。对于del（5q）亚型的症状性贫血，治疗首选来那度胺。来那度胺可使部分患者减轻或脱离输血依赖，并获得细胞遗传学缓解，生存期延长。来那度胺用量为：10 mg/d，连用21 d，每28天为1个疗程，持续治疗2～4个月以评估疗效。来那度胺治疗3～6个月后HGB升高<15 g/L或红细胞输注依赖无改善，提示治疗无效。来那度胺最常见的≥3级不良反应为中性粒细胞减少和血小板减少。因此，10 mg/d×21 d方案适用于PLT>50×10^9^/L及ANC>0.5×10^9^/L的患者。对于伴有del（5q）但无输血依赖的较低危MDS患者，5 mg/d的小剂量来那度胺可降低患者发生输血依赖的风险，80％患者达到细胞遗传学反应，70％患者获得细胞遗传学的完全缓解（CR）。伴有del（5q）的MDS患者如出现下列情况不建议应用来那度胺：①骨髓原始细胞比例>5％；②合并复杂染色体核型或染色体−7；③较高危组MDS；④合并TP53基因突变。对于上述情况，推荐采用较高危组MDS的治疗方案。来那度胺在其他亚型中的应用详见下文。

3. 免疫抑制治疗（IST）：IST包括抗胸腺细胞球蛋白（ATG）±环孢素A，可考虑用于具备下列特征的较低危患者：年龄≤60岁、骨髓原始细胞比例≤5％、低增生MDS、正常核型或单纯染色体+8、存在输血依赖、HLA-DR15阳性、阵发性睡眠性血红蛋白尿症克隆阳性及合并STAT3突变。环孢素A起始剂量为3～5 mg·kg^−1^·d^−1^，空腹血药浓度维持在100 µmol/L以上，持续6个月。ATG剂量参考再生障碍性贫血用法。对于合并血小板减少患者，可采用IST±TPO-RA。

4. 症状性贫血伴del（5q）亚型：若血清促红细胞生成素（sEPO）浓度≤500 mU/ml，首先推荐来那度胺，其次推荐hEPO。若上述治疗无效，治疗参考下文中sEPO浓度>500 mU/ml但来那度胺疗效不佳患者的治疗路径。

若sEPO浓度>500 mU/ml，首先推荐来那度胺治疗。若疗效不佳，患者合并IST使用特征时，可采用IST±TPO-RA。以上治疗效果欠佳的患者建议采用小剂量去甲基化治疗、参加临床试验或接受异基因造血干细胞移植（allo-HSCT），也可根据异柠檬酸脱氢酶1（IDH1）基因突变状态决定是否联合艾伏尼布治疗。

5. 症状性贫血伴SF3B1突变亚型：首选罗特西普治疗。若罗特西普疗效不佳，根据sEPO浓度决定治疗方案。若sEPO浓度≤500 mU/ml，推荐hEPO±G-CSF。若sEPO浓度>500 mU/ml，建议接受来那度胺治疗。若以上治疗均无明显应答，考虑IST或依据IDH1基因突变状态决定是否联合艾伏尼布靶向治疗。若以上治疗应答仍不佳，建议参加临床试验或接受allo-HSCT。

6. 症状性贫血伴非del（5q）、非SF3B1突变亚型：若sEPO浓度≤500 mU/ml，首先推荐采用hEPO治疗，也可采用罗特西普治疗。其他推荐包含hEPO+G-CSF、来那度胺、hEPO+来那度胺。若sEPO浓度>500 mU/ml，可根据疾病特征接受IST±TPO-RA。也可尝试hEPO联合ATRA及十一酸睾酮三药组合。建议参加临床试验、接受去甲基化治疗、接受罗特西普治疗。若效果仍不佳，可根据IDH1基因突变状态决定是否联合艾伏尼布靶向治疗或allo-HSCT。

7. 症状性血小板减少或中性粒细胞减少：优先考虑临床试验。也可尝试小剂量地西他滨（Decitabine，DAC）或阿扎胞苷（Azacitidine，AZA）治疗，具体为：DAC 20 mg·m^−2^·d^−1^×3 d或AZA 75 mg·m^−2^·d^−1^×3 d，每4周为1个疗程。适合接受IST的患者，也可考虑接受IST±TPO-RA治疗。严重的单一血小板减少患者可考虑单独应用TPO-RA，但需警惕MDS白血病转化风险。对于存在IDH1基因突变的患者，可联合艾伏尼布分子靶向治疗。若以上治疗无效或发生疾病进展，考虑其他临床试验或allo-HSCT。

（三）较高危组MDS的治疗

较高危组包含IPSS评分>1分的中危-1组/高危组、IPSS-R评分>3.5分的中危组/高危组/极高危组及IPSS-M评分>0分的中高危组/高危组/极高危组。较高危组MDS的治疗目标是延缓疾病进展、延长患者生存期甚至治愈，其治疗路径见图2。

**图2:**
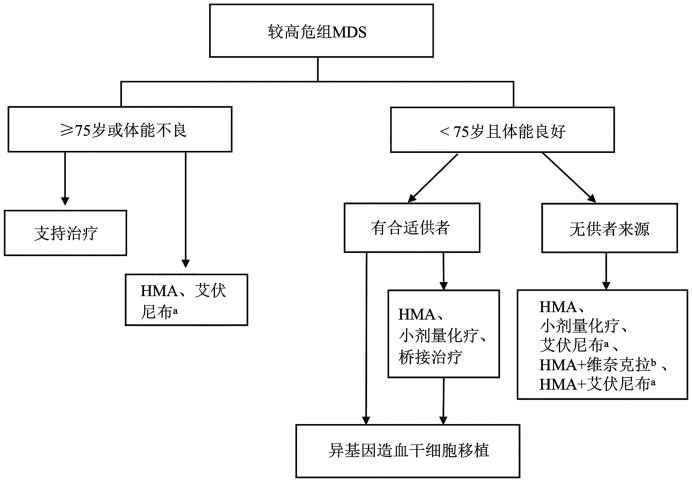
较高危组骨髓增生异常肿瘤（MDS）的治疗路径 **注** HMA：去甲基化治疗；^a^艾伏尼布仅适用于伴IDH1突变患者；^b^HMA+维奈克拉用于复发难治患者或移植前桥接治疗

1. 去甲基化治疗：推荐用于不适合造血干细胞移植的较高危MDS患者。与支持治疗组相比，去甲基化药物治疗组可降低患者向AML进展的风险、改善患者的生存。常用药物包括AZA及DAC。AZA及DAC的有效性及安全性也在国内多中心的注册研究中得到证实[16]–[17]。

（1）AZA：推荐用法为75 mg·m^−2^·d^−1^×7 d，皮下注射，每4周为1个疗程。接受AZA治疗的MDS患者，首次获得治疗反应的中位时间为3个疗程，约90％治疗有效的患者在6个疗程内获得治疗反应。因此，推荐MDS患者接受至少6个疗程AZA后评价治疗反应，有效患者可持续使用。

（2）DAC：推荐用法为20 mg·m^−2^·d^−1^×5 d，每4周为1个疗程，至少接受4个疗程后评价治疗反应，有效患者可持续使用。

（3）其他联合治疗方案：已有研究报道，接受ATRA联合DAC治疗的较高危MDS患者的总有效率、CR率及无进展生存率均显著高于接受DAC单药治疗患者[18]。

2. AML样治疗及小剂量化疗：AML样治疗适用于年龄<70岁、体能良好、骨髓原始细胞比例≥10％患者的移植前桥接治疗，也适用于不适合移植的较高危患者或复发难治患者。国内常用的治疗方案为VA方案，具体为：AZA 75 mg/m^2^，第1～7天；维奈克拉100 mg第1天，200 mg第2天，自第3天开始400 mg，应用时间依据血象调整，一般应用7～14 d。最新的Ⅲ期临床试验对比AZA联合维奈克拉与AZA单药治疗高危MDS的疗效，患者的OS率无明显差异。对于复发难治患者，鼓励参加临床试验。小剂量化疗方案为：阿糖胞苷20 mg·m^−2^·d^−1^，连用10～14 d，每4周为1个疗程。对于存在预后不良染色体核型的患者，小剂量化疗的疗效劣于AZA去甲基化治疗。

3. 分子靶向治疗：

（1）BCL-2抑制剂：新型靶向药物B细胞淋巴瘤2（BCL-2）抑制剂维奈克拉与去甲基化治疗或靶向IDH1抑制剂联合用于高危难治MDS患者，在前期的临床试验中表现出良好的效果。在一项应用AZA联合维奈克拉治疗高危MDS的Ⅰb期临床试验中，维奈克拉的用量为400 mg每日1次×14 d；AZA 75 mg·m^−2^·d^−1^×7 d，每4周为1个疗程。纳入分析的107例患者中，29.9％达CR，中位OS期为26个月[19]。对于高危MDS，一项Ⅲ期临床试验表明，患者接受AZA联合维奈克拉治疗的OS率较接受AZA单药治疗无显著提高。鼓励患者参加包含维奈克拉的临床试验，但需密切关注药物相关的骨髓抑制。维奈克拉最常见的≥3级不良反应是中性粒细胞减少和血小板减少。

（2）IDH1突变抑制剂：约4％的MDS患者存在IDH1基因突变。IDH1突变抑制剂艾伏尼布在国内已获批治疗伴IDH1突变的复发难治AML[20]。一项Ⅰ期试验评估了艾伏尼布对伴IDH1基因突变的复发难治MDS患者疗效和安全性。共入组19例患者，纳入疗效评估的18例患者中，艾伏尼布的CR率+部分缓解率为38.9％。达CR患者中，68.6％患者缓解持续时间超过5年，中位OS期35.7个月。71.4％需要输注红细胞及75.0％需要输注血小板的患者脱离了输血依赖。2024年FDA批准艾伏尼布治疗伴IDH1突变的复发难治MDS。艾伏尼布常见的≥3级不良反应为疲劳和低钠血症。目前主要用于临床试验，可与去甲基化治疗联合应用，常见的不良反应主要为骨髓抑制，包括中性粒细胞减少、血小板减少、贫血等。

4. allo-HSCT：allo-HSCT是目前唯一能根治MDS的治疗手段，造血干细胞来源包括同胞全相合供者、HLA匹配的无关供者、单倍型相合的供者或脐带血。allo-HSCT的适应证为：①年龄<75岁、造血干细胞移植合并症指数（HCT-CI）评分0～2分、Karnofsky体能状态（KPS）评分≥80％，MDS特异性衰弱指数（FI）评分<0.3的较高危组MDS患者；②年龄<75岁、伴严重血细胞减少、其他治疗无效的较低危组患者。

近年研究发现，胚系突变如CEBPA、DDX41、TP53、RUNX1、ANKRD26、ETV6、GATA2、SRP72、SAMD9、SAMD9L、BLM等可导致发生MDS及AML的风险升高。对于拟行移植的MDS患者，若存在以下特征之一：确诊年龄<50岁、合并≥1种其他肿瘤、体细胞突变合并潜在致病性胚系突变、疑似家族性肿瘤综合征患者，应对供者及受者的胚系突变进行检测，从而优化供者选择。移植前若骨髓原始细胞比例≥10％，是否行桥接治疗（去甲基化治疗、化疗或分子靶向治疗）仍有争议。对于年龄≤55岁的移植患者，推荐清髓性预处理方案。对于年龄>55岁，一般状态或脏器功能差的患者可使用减低剂量或减低毒性的预处理方案。对于较高危患者，尤其是IPSS-M或IPSS-R评分高危或极高危患者，尽早移植能给患者带来生存获益。MDS合并TP53突变，尤其是双等位基因失活患者，即使接受移植，总体预后也很差。若桥接治疗后骨髓原始细胞比例仍>5％，不应为降低肿瘤负荷而耽误移植的进行。

六、疗效评估

MDS国际工作组（International Working Group，IWG）于2000年提出国际统一疗效标准，2006年进行了修订，2018年和2023年分别对血液学改善和高危MDS的疗效评估标准进行更新[21]–[24]。2023版IWG标准考虑了最新的MDS分子评分系统IPSS-M，强调了细胞遗传学缓解在疗效评估中的重要性，首次提出等同于CR（CR equivalent）标准，剔除了原标准中的骨髓CR（mCR）标准，并对MDS治疗后血象恢复程度有了更精细化的分层评估。新标准同时剔除了原标准中的疾病稳定（SD）及无病生存（DFS）。2006版及2023版IWG评估标准见表9[24]。

**表9:** 骨髓增生异常肿瘤（MDS）国际工作组（IWG）疗效评估标准

疗效	IWG 2006（全部MDS患者）	IWG 2023（高危MDS患者）
完全缓解（CR）	骨髓：原始细胞比例≤5％，可伴有持续存在的血细胞发育异常； 外周血：血红蛋白（HGB）≥110 g/L，中性粒细胞绝对计数（ANC）≥1.0×10^9^/L，血小板计数（PLT）≥100×10^9^/L，原始细胞为0	骨髓：原始细胞比例<5％^a^，可伴有持续存在的血细胞发育异常； 外周血：HGB≥100 g/L，ANC≥1.0×10^9^/L，PLT≥100×10^9^/L，原始细胞为0^b^
等同于完全缓解（CR equivalent）	–	适用于基线或治疗前骨髓原始细胞比例<5％的较高危患者 骨髓：原始细胞比例<5％^a^，可伴有持续存在的血细胞发育异常； 外周血：HGB≥100 g/L，ANC≥1.0×10^9^/L，PLT≥100×10^9^/L，原始细胞为0^b^； 基线存在的细胞遗传学异常完全缓解
形态学缓解（mCR）	骨髓：原始细胞比例≤5％且较治疗前原始细胞减少≥50％ 外周血：无要求	剔除
部分缓解（PR）	其他条件均达到CR标准，骨髓原始细胞比例较治疗前减少≥50％，但绝对值仍>5％；不考虑骨髓细胞增生程度和形态学变化	其他条件均达到CR标准，骨髓原始细胞比例较治疗前减少≥50％，但绝对值仍≥5％； 不考虑骨髓细胞增生程度和形态学变化
疾病稳定（SD）	未达到PR的最低标准但超过8周无疾病进展证据	剔除
CR伴有限的血细胞恢复（CR_L_）^c^CR_L_=CRuni+CRbi	–	骨髓：原始细胞比例<5％^a^，可伴有持续存在的血细胞发育异常； 外周血：原始细胞为0^b^； CRuni：外周血细胞计数未达到CR标准，但符合下列1项：HGB≥100 g/L，ANC≥1.0×10^9^/L，PLT≥100×10^9^/L； CRbi：外周血细胞计数未达到CR标准，但符合下列2项：HGB≥100 g/L，ANC≥1.0×10^9^/L，PLT≥100×10^9^/L
CR伴部分血细胞恢复（CRh）^c^	–	骨髓：原始细胞比例<5％^a^，可伴有持续存在的血细胞发育异常； 外周血未达到CR或CR_L_的最低标准，但满足ANC≥0.5×10^9^/L，PLT≥50×10^9^/L，原始细胞为0^b^；对HGB水平无要求
血液学改善（HI）	–	–
红系反应（HI-E）	–	–
输血依赖分类标准^d^	分2组（仅适用于HGB<90 g/L者）： 1. 无输血依赖：8周内红细胞输注<4个单位； 2. 有输血依赖：8周内红细胞输注≥4个单位	分3组： 1. 无输血依赖（NTD）：持续16周未输注红细胞； 2. 低输血负担（LTB）：16周内至少输血2次，总计输注3～7个单位红细胞；或8周内最多输血3次，总计3～7个单位红细胞； 3. 高输血负担（HTB）：16周内输注≥8个单位红细胞或8周内输注≥4个单位红细胞
无输血依赖（NTD）	1. 治疗前HGB<110 g/L纳入红细胞反应评估； 2. 仅治疗前HGB≤90 g/L且需要红细胞输注者才纳入红细胞输注疗效评估； 3. 治疗后HGB升高≥15 g/L，或与治疗前8周比较，每8周红细胞输注量至少减少4个单位	在16～24周的观察期内，与治疗开始前16周内2次HGB（除输血外）的最低平均值相比，至少有2次连续HGB增加≥15 g/L，且持续时间至少为8周，一般认为至少持续16周才具有临床意义
低输血负担（LTB）	–	16～24周的观察期内，与治疗前16周相比，至少有8周没有输血，一般认为至少16周无输血才具有临床意义
高输血负担（HTB）	–	主要应答：16～24周的观察期内，与治疗前16周相比，至少有8周没有输血，一般认为至少16周无输血才具有临床意义 轻微应答：与治疗前16周相比，16周内红细胞输注减少≥50％
血小板反应（HI-P）	1. 疗效必须维持超过8周，仅评估治疗前PLT<100×10^9^/L的患者； 2. 治疗前PLT>20×10^9^/L，治疗后PLT增加≥30×10^9^/L； 3. 若治疗前PLT<20×10^9^/L，治疗后PLT>20×10^9^/L且PLT至少增加1倍	1. 治疗前PLT>20×10^9^/L且<100×10^9^/L，治疗后PLT增加≥30×10^9^/L； 2. 治疗前PLT<20×10^9^/L，治疗后PLT>20×10^9^/L且PLT至少增加1倍； 3. 考虑患者的出血症状变化； 4. 若治疗前PLT>100×10^9^/L，PLT增加的数值也要报告
中性粒细胞反应（HI-N）	1. 评估仅限于治疗前ANC<1.0×10^9^/L患者； 2. 治疗后ANC增加1倍以上且绝对值增加>0.5×10^9^/L	1. 治疗前ANC<1.0×10^9^/L，治疗后ANC增加1倍以上且绝对值增加>0.5×10^9^/L； 2. 治疗前ANC>1.0×10^9^/L者，治疗后ANC的增加也要报告
总有效率（ORR）	–	ORR=CR（或CR equivalent）率+PR率+CR_L_率+CRh率+HI率
无效	–	未达到CR、CR equivalent、PR、CR_L_、CRh或HI标准
无法评估疗效	–	临床试验中患者可能有效但因早期死亡、早期退组、骨髓标本无法获得或标本质量不达标而无法评估
细胞遗传学反应	CR：染色体异常消失且无新发异常； PR：染色体异常细胞比例减少≥50％	CR：染色体异常消失且无新发异常； PR：染色体异常细胞比例减少≥50％
疾病进展（PD）^e^	1. 治疗前原始细胞比例<5％，治疗后原始细胞增加≥50％且绝对值>5％； 2. 治疗前原始细胞比例5％～10％，治疗后原始细胞增加≥50％且绝对值>10％； 3. 治疗前原始细胞比例10％～20％，治疗后原始细胞增加≥50％且绝对值>20％； 4. 治疗前原始细胞比例20％～30％，治疗后原始细胞增加≥50％且绝对值>30％； 5. 符合下列标准之一： ①ANC或PLT较最佳疗效时减少≥50％； ②HGB减少≥20 g/L； ③出现输血依赖	符合下列标准之一： 1. 因原始细胞细胞增多导致的PD：原始细胞增加≥50％，且绝对值较治疗前增加≥5％； 2. 因血细胞减少恶化导致的疾病进展：在8周内出现新发的、多次需要输注红细胞或血小板，与急性并发症（如败血症、消化道出血）或治疗效果无关，且未达到HI的最低标准； 3. 进展为急性髓系白血病：原始细胞较基线增加≥50％且绝对值≥20％
疾病复发	符合下列至少1项： 1. 骨髓原始细胞回升至治疗前水平； 2. ANC或PLT较最佳疗效时下降≥50％； 3. HGB下降≥15 g/L或出现输血依赖	1. 因原始细胞细胞增多导致的疾病复发：原始细胞较前增加≥50％，且绝对值达到5％以上；外周血再次出现原始细胞；或出现髓外浸润（如粒细胞肉瘤）； 2. 因血细胞减少恶化而导致的疾病复发：PLT或ANC较最佳疗效时下降≥50％或HGB下降≥15 g/L，且伴有同系血细胞计数绝对值降低，要求HGB<100 g/L、PLT<100 ×10^9^/L，或ANC<1.0×10^9^/L，或需反复（1次以上且间隔时间≥1周）输注红细胞或血小板，且此输血依赖与急性并发症（如败血症、消化道出血）或治疗效果无关。此外，外周血细胞变化未达到任何一种HI标准
HI后的进展或复发	至少符合下列标准之一： 1. ANC或PLT较最佳疗效时下降≥50％； 2. HGB下降≥15 g/L； 3. 依赖输血	–
治疗失败	治疗期间死亡，或病情进展表现为血细胞减少加重、骨髓原始细胞增加或较治疗前发展为更晚期的FAB亚型	–
总体生存（OS）	终点：任何原因的死亡	从临床研究开始时间至因任何原因死亡的时间
无事件生存（EFS）	终点：治疗失败或因任何原因死亡	从临床研究开始至出现下列任何一种情况的时间： 1. PD； 2. 研究开始后6个月未达到CR、CR equivalent、PR、CR_L_、CRh或HI标准； 3. 已经达到CR、CR equivalent、PR、CR_L_、CRh或HI标准，出现疾病复发； 4. 因任何原因死亡
无进展生存（PFS）	终点：病情进展或因任何原因死亡	从临床研究开始至出现下列任何一种情况的时间： 1. PD； 2. 已经达到CR、CR equivalent、PR、CR_L_、CRh或HI标准，出现疾病复发； 3. 因任何原因死亡
无病生存（DFS）	终点：疾病复发或因任何原因死亡	剔除

**注** –：无内容；CRuni：CR伴一系外周血细胞恢复；CRbi：CR伴两系外周血细胞恢复。^a^治疗前原始细胞≥5％，才具备评估CR、PR、CRh或CR_L_的条件。若通过外周血细胞计数评估疗效，其时间窗口以骨髓检查的前后2周作为参考。对于原始细胞<5％，因不良细胞遗传学或严重血细胞减少而确定的高危MDS，细胞遗传学完全清除（完全细胞遗传学反应）和血细胞计数符合CR标准被视为等同于CR，应单独报告为CR equivalent。全血细胞计数完全恢复指HGB≥100 g/L、PLT≥100×10^9^/L、ANC≥1.0×10^9^/L，与基线外周血计数水平无关。鉴于分子清除率尚未经过前瞻性验证，因此未将其用于CR的定义。^b^外周血原始细胞与骨髓活检中的原始细胞可能存在差异（原始细胞比例：0～5％）。在此情况下，应在2周内再次复查外周血涂片，以区分原始细胞升高是否与疾病相关（如在骨髓恢复期、发生感染等情况）。如在骨髓活检后2周内，外周血原始细胞为0，且骨髓活检显示原始细胞比例<5％，则患者已达到CR，无需再次进行骨髓活检确认。^c^CR_L_和CRh是临时定义，需要额外的前瞻性研究验证。与CR和PR相似，两者均由疗效评估前后的血细胞计数决定，与基线血细胞计数无关。若要符合CR_L_的条件，患者需要在评估时或评估前后一系或两系外周血细胞计数水平达到或超过特定血细胞的CR阈值。对于2022年国际共识分类和第5版WHO分类分别定义的MDS/急性髓系白血病（AML）或MDS原始细胞增多患者，可考虑报告CRh，其定义为骨髓原始细胞<5％，外周血原始细胞为0，外周血细胞计数部分恢复（PLT≥50×10^9^/L，ANC≥0.5×10^9^/L），以实现与欧洲白血病网络（ELN）2022 AML反应标准一致。如患者同时符合CR_L_和CRh的标准，则在ORR中应报告为达到CR_L_，因为CR_L_代表了更高的血液学改善阈值。^d^关于评估输血依赖或脱离输血依赖的筛选期和时间窗，筛选期以前16周外周血HGB来评估；考虑高危MDS的严重性，筛选前8周的HGB水平也可以。^e^建议采用骨髓活检评估疾病进展。对于需要输血支持的疾病进展或复发患者，以首次输注红细胞和血小板的日期为疾病进展日期。克隆进展定义为获得新的细胞遗传学或分子异常，可作为临时疾病进展标准进行报告。除非方案另有规定，否则并不一定构成临床进展。1个单位红细胞通常来自200～250 ml全血
